# Effector function of CTLs is increased by irradiated colorectal tumor cells that modulate OX-40L and 4-1BBL and is reversed following dual blockade

**DOI:** 10.1186/s13104-016-1914-9

**Published:** 2016-02-13

**Authors:** Anita Kumari, Charlie Garnett-Benson

**Affiliations:** Department of Biology, Georgia State University, 161 Jesse Hill Jr. Dr, Atlanta, GA 30303 USA

**Keywords:** Ionizing radiation, Tumor immunity, Cytotoxic T cells, Immunogenic modulation, Effector costimulation, Colorectal

## Abstract

**Background:**

Sub-lethal doses of ionizing radiation (IR) can alter the phenotype of target tissue by modulating genes that influence effector T cell activity. Previous studies indicate that cancer cells respond to radiation by up-regulating surface expression of death receptors, cell adhesion molecules and tumor-associated antigens (TAA). However, there is limited information available regarding how T cells themselves are altered following these interactions with irradiated tumor cells.

**Methods:**

Here, several human colorectal tumor cell lines were exposed to radiation (0–10 Gy) in vitro and changes in the expression of molecules costimulatory to effector T cells (4-1BBL, OX-40L, CD70, ICOSL) were examined by flow cytometry. T cell effector function was assessed to determine if changes in these proteins were directly related to the changes in T cell function.

**Results:**

We found OX-40L and 4-1BBL to be the most consistently upregulated proteins on the surface of colorectal tumor cells post-IR while ICOSL and CD70 remained largely unaltered. Expression of these gene products correlated with enhanced killing of irradiated human colorectal tumor cells by TAA-specific T-cells. Importantly, blocking of both OX-40L and 4-1BBL reversed radiation-enhanced T-cell killing of human tumor targets as well as T-cell survival and activation.

**Conclusions:**

Overall, results of this study suggest that, beyond simply rendering tumor cells more sensitive to immune attack, radiation can be used to specifically modulate expression of genes that directly stimulate effector T cell activity.

## Background

Radiotherapy (RT) is an extremely common modality in cancer treatment. Many cancer patients undergo RT during their course of illness however tumor cells acquire mutations during development that inhibit cell death by radiation [1]. RT also fails to control systemic disease and many cancer patients experience disease recurrence [2]. Moreover, radiation has significant dose limiting toxicities when used as a definitive therapy or in sensitive tissues such as the colon [3, 4]. Cancer immunotherapy (CIT) is emerging as an attractive therapeutic option and many standard cancer therapies, such as chemotherapy and radiation, rely on induction of functional immune cells for efficacy [5, 6]. Indeed, combination CIT and RT is more effective in treating metastatic and reoccurring cancers than either of the therapies alone [7–15]. Fully understanding the role of RT in tumor immunity will have a major impact on the treatment of cancers combining these modalities [16].

Effective cancer immunotherapy (CIT) strategies aim to generate tumor-specific CD8+ CTLs to attack and kill tumor cells [17, 18]. To elicit an effective immune response against tumors, the immune system needs to recognize tumor-associated antigen (TAA) presented to the TCR within MHC Class-I molecules, in conjunction with appropriate co-stimulation [19, 20]. Most cancer patients have some level of TAA-specific T cells. Surprisingly, treatments that further induce or expand the number of anti-TAA CTLs do not consistently translate into objective clinical tumor responses. As such, it is clear that that increasing tumor-specific CTL numbers is insufficient to control malignant cells and eliminate cancer. Several possibilities for this outcome include weak immunogenicity of TAA, low expression of co-stimulatory molecules on tumor cells and APCs, and/or secretion of suppressive molecules or recruitment of suppressive cells.

Local tumor irradiation has been shown to generate tumor-specific CTL and enhance anti-tumor immune responses [21–25]. Sub-lethal doses of ionizing radiation (IR), for example, have been reported to up-regulate expression of immune-stimulatory proteins in various tissue types both in vitro and in vivo [25, 26]. We previously reported that exposure of human carcinoma cell lines to sub-lethal radiation results in enhanced susceptibility to lysis by tumor specific cytotoxic T cells (CTLs) [26, 27]. Significantly enhanced killing by CEA-specific CD8+ CTLs was observed in five of five colorectal carcinoma (CRC) cell lines exposed to a single dose of 10 Gy radiation. Furthermore, enhanced attack by CTLs in head and neck squamous cell carcinoma [27] and prostate carcinoma [28] suggests the functional enhancement is not limited to a single antigen-specificity or cancer type. More recently we reported that irradiation of human tumor cells imparts enhanced and sustained susceptibility to multiple death receptor signaling pathways [29]; however, the differences in magnitude of lysis among the cell lines does not correlate with altered expression of death receptors, nor altered surface expression of MHC-I, ICAM-1 or TAAs [26]. Thus, the mechanism of enhanced CTL killing against human carcinoma cells is unclear and surprisingly few studies focus on understanding the effect of radiation-induced changes in tumor cells on CTL effector activity and function.

Tumor derived antigens often induce insufficient co-stimulation and induce immune tolerance to the antigen. Antigen presentation in a toleragenic or immunosuppressive environment where robust costimulation is not present leads to sub-optimal immune responses such as T-cell anergy. T-cell co-stimulatory agonists can program T cells encountering these non-immunogenic antigens to expand and develop anti-tumor effector activities [30]. As a result, strategies for improving positive co-stimulation to T cells and reversing negative regulation of T cells are currently very attractive therapeutic approaches for cancer therapy. The latter approach has resulted in recent FDA approval of several T cell checkpoint blockade agents. Regarding positive signals to T cell, the co-stimulatory molecules 4-1BB ligand (4-1BBL/TNFSF9/CD137L) and OX-40 ligand (OX-40L/TNFSF4/CD134L/CD252) are important regulators of CTL function, and lack of signaling through these molecules results in reduced CTL activity [20, 31–33]. In tumor bearing mice, intratumoral OX-40 activation increases CD40 expression on T cells and increases the effector memory T cells (T_EM_) subset [34]. 4-1BBL (TNFRSF9/CD137) costimulation of tumor-specific T cells is important for T-cell activation and 4-1BBL transfected DCs elicit more effective responses and enhanced CTL killing of tumor cells, due to increased expression of perforin and IFN-γ [35]. In recognition of the importance of these pathways to generating effective antitumor immunity, clinical studies have started to evaluate the effectiveness of humanized agonist antibodies to both OX-40 and 4-1BB [36–39]. Engagement of OX-40 and 4-1BB by agonist (activating) antibodies increases tumor immunity, resulting in long-term survival in a number of murine tumor models [32, 40–42]. These costimulatory signals may be particularly important for effective responses against self-antigen such as those expressed by many tumor cells. In the absence of these co-stimulatory signals anti-tumor effector T-cells may be rendered anergic.

Our previous studies suggest that sub-lethal doses of radiation cause altered expression of genes within tumor cells resulting in increased CTL-mediated lysis [26]. More recently, we reported that radiation increased expression both OX-40L and 4-1BBL in human prostate cancer cells [43], and that increased expression of 4-1BBL in colorectal tumor cells occurred via epigenetic changes at the promoter [44]. The present study was designed to test the hypothesis that enhanced activity of TAA-specific CTLs against tumor cells surviving radiation is mediated, in part, through increased effector co-stimulation from OX-40L and 4-1BBL on tumor cells. To our knowledge, this is the first study to (a) demonstrate that radiation specifically modulates OX-40L and 4-1BBL expression while leaving expression of other co-stimulatory molecules such as CD70 and ICOSL unchanged, (b) report OX-40L and 4-1BBL expression upregulated in a panel of colorectal cancer cell lines post-IR, (c) show that irradiated tumor cells that do not increase co-stimulatory molecule expression also do not increase T cell activity, and (d) determine that CTL killing of irradiated tumor cells is abolished in the presence of a neutralizing antibody against OX-40L and silenced 4-1BBL expression. Overall, the results of this study suggest that tumors surviving radiation therapy are not simply rendered more ‘sensitive to T cells attack’ but actively modulate expression of proteins that induce enhanced effector CTL function and activity.

## Methods

### Cell lines

Human colorectal carcinoma cell lines HCT116, Caco-2 and WiDr, were obtained from the laboratory of tumor immunology and biology, LTIB, NCI, NIH. SW620, HT-29, LS174T and Colo205 cells were purchased from ATCC. All cells were cultured as recommended by ATCC and tested periodically to ensure absence of Mycoplasma. Cells were incubated at 37 °C incubator with 5 % CO2. The use of these de-identified and commercially purchased cell lines received exempt approval under a human investigation protocol approved by the Institutional Review Board of Georgia State University (#H13305).

### Irradiation

A RS-2000 biological X-ray irradiator (Rad source technology, Suwanee, GA) was used to irradiate tumor cells. Cells were irradiated at a dose rate of 2 Gy/min at voltage and current of 160 kV and 25 mA, respectively. Cells were maintained in suspension and kept on ice during irradiation. Immediately after irradiation, cells were centrifuged and cells were plated in tissue culture plates in fresh media.

### RNA isolation

At 24 or 48 h post-IR, RNA was extracted from tumor cells using RNeasy mini kit (Qiagen Inc. Valencia, CA) according to manufacturer’s instructions. Purified RNA was DNase-treated by Rnase-free DNase (Qiagen Inc. Valencia, CA) following manufacturer’s instructions.

### Flow cytometry

Cells were stained with species-specific primary labeled mAb [CD137L (4-1BBL)-PE, CD252 (OX-40L)-PE, CD70-FITC, ICOSL-PE, CD8α-FITC, CD107a-APC, CD25-APC, CD69-PE, CD66-PE, CD227 (MUC-1)-FITC] purchased from BioLegend or BD biosciences (San Diego, CA). Surface staining was done in cell staining buffer for 30 min on ice. 7AAD dye were obtained from BD biosciences (San Diego, CA) and used according to manufacturers instructions to measure cell death. Intracellular staining of active caspase-3 was done according to manufacturer’s instruction. Flow cytometry data were acquired on BD Fortessa and analyzed with FlowJo software (TreeStar). The live cells population was gated on the FSC and SSC scatter plots for analysis of surface proteins. No live cells gate was used for cell death analysis samples. Samples were stained with the appropriate isotype control antibodies and gates were set to less than 5 % in all isotype control samples.

### Generation of cytotoxic T-lymphocytes

Cell-rich leukapheresis collections from HLA-A2+ donors were obtained from Hemacare (Van Nuys, CA), with appropriate informed consent, for generating antigen specific CTLs as previously described [27, 45, 46]. These commercially purchased (and de-identified) tissues received exempt approval under a human investigation protocol approved by the Institutional Review Board of Georgia State University (#H13305). Briefly, PMBCs that adhered to the tissue culture flask after 2 h were cultured for 7 days in AIM-V media (Invitrogen) containing 100 ng/ml of human granulocyte colony stimulating factor (GM-CSF) and 20 ng/ml of IL-4 (Miltenyi Biotec, Inc. Auburn, CA). On the fifth day in culture 500 ng/ml CD40L (Millipore corporation, Temecula, CA) was added to mature the DCs. Matured DCs were then collected and loaded with 40 μg/mL of HLA-A2 binding peptides. CEA peptide (YLSGANLNL (CAP-1; [46]) or MUC (ALWGQDVTSV) peptides were allowed to bind to the DCs for 4 h in a 37 °C 5 % CO_2_ incubator. and subsequently irradiated with 50 Gy. Immunomagnetic beads (Miltenyi Biotec Inc. Auburn, CA) were used to isolate CD8+ T cells from the non-adherent fraction of PBMCs. The peptide pulsed DCs were used to stimulate CD8+ T cells in media containing 10 ng/ml of IL-7 (Millipore, Temecula, CA). After 3 days in culture 30U/ml of IL2 (Millipore, Temecula, CA) were added. T-cells were restimulated in this manner weekly using autologous antigen presenting cells. On the fourth day of stimulation T cells were isolated and used in a standard cytotoxic killing assay.

### Cytotoxicity assay

CTL lysis of HCT116, SW620, Colo205, and LS174T (HLA-A2-) tumor cells was measured using the DELFIA cell cytotoxicity kit (Perkin Elmer). Seventy two hour after irradiation, viable and proliferating tumor cells (2 × 10^6^/2 ml) were harvested, counted, and incubated with 5 μl of BATDA (bis (acetoxymethyl) 2,2′:6′,2′′- terpyridine- 6,6′′- dicarboxylate; PerkinElmer, Boston, MA) for 20 min at 37 °C. After incubation, cells were washed four times with PBS. 5 × 10^3^ cells were added in triplicate to a 96-well U-bottom plate, and either CEA or MUC specific CD8+ T-cell were added to the wells (E:T ratios between 12:1 and 30:1) and incubated for 4–5 h at 37 °C. After incubation, the plate was centrifuged (500*g* for 5 min) and 20 μl of supernatant were transferred into a flat bottom plate. Two hundred microlitre of Europium solution was added and incubated for 15 min at room temperature on plate shaker [47]. Lysis was measured on a time resolved Victor3 plate reader fluorometer. The percentage of tumor lysis was calculated as follows:  % tumor lysis = experimental release (counts) − spontaneous release (counts)/maximum release (counts) − spontaneous release (counts) × 100.

### Expression knock-down and blocking

4-1BBL gene expression was knocked down using a gene specific siRNA. Briefly, tumor cells were plated in a 6-well dish at 1 × 10^5^ cells/well 1 day prior to transfection, with 50–70 % confluence on the day of transfection. In some experiments 2 × 10^4^ cells were plated in 24-well plates. 4-1BBL Flexi Tube siRNA #6 (Qiagen Inc. Valencia, CA) was diluted in optiMEM medium (invitrogen) and transfected using Hyperfect (Qiagen Inc. Valencia, CA). Twenty-four hours post transfection; cells were irradiated with 10 Gy or mock-irradiated. The cells were harvested 24–48 h post irradiation and 4-1BBL mRNA expression was measured. A negative control siRNA that was not specific to 4-1BBL was also transfected into cells and 4-1BBL mRNA similarly evaluated. Using combination 4-1BBL and OX-40L siRNA to knock down both genes simultaneously resulted in incomplete knock-down of both genes in our tumor cells. As a result, for dual blockade experiments, we knocked down 4-1BBL using siRNA and we used a Goat anti-human OX-40L-neutralizing antibody (R&D system, Minneapolis, MN) to block OX-40 ligand and receptor interaction (cat #: AF1054). In the indicated groups, 500 ng/ml of anti-human OX-40L neutralizing antibody was added to Eu-labeled tumor cells for 15 min prior to adding TAA-specific CTLs. The anti–human 4-1BB monoclonal blocking antibody BBK-2 [48] was added 20 μg/ml 15 min before T-cells were added. Isotype matched antibodies were added to the other groups as a negative control. In parallel experiments, the percent of T cells expressing CD25 (activation) or positive for active Caspase-3 (cell death) was measured by flow-cytometry as previously described [44].

### Statistical analysis

Statistical difference in the distribution of flow cytometric data from several repeat experiments were graphed and the mean of three to four independent experiments were calculated and an un-paired two-tailed student T-test was performed using Graphpad by Prism. Statistical differences between groups in the cytolysis assays, activation, and survival assays were calculated using un-paired one or two-tailed student T-test and calculated for the 95 % confidence interval (CI).

## Results and discussion

### Sub-lethal irradiation of colorectal carcinoma cell lines does not modulate all T cell stimulatory molecules the same

**Fig. 1:**
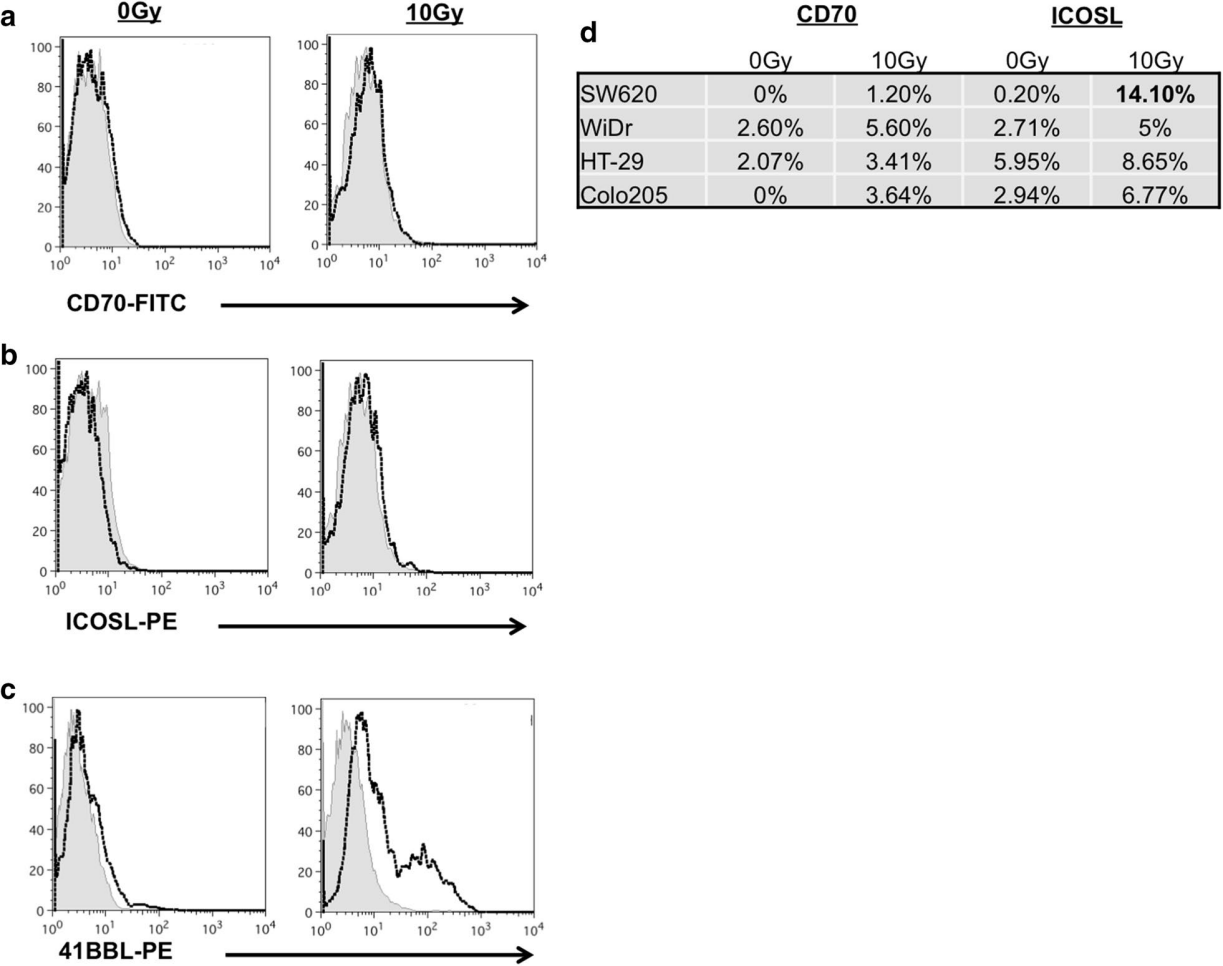
Expression of CD70 and ICOSL following tumor cell irradiation. **a** CD70 expression (*black line*). **b** ICOSL expression (*black line*). **c** 4-1BBL expression (*black line*) 48 post-IR in HCT116 cells. Isotype control stained cells were all less than 5 % positive (*filled grey histogram*). **d** CD70 and ICOSL expression in treated and untreated SW620, WiDr, HT-29 and Colo205 cells. Experiments repeated two and three times with similar results

There are a number of proteins that, when expressed by target cells, can contribute to enhanced local activity of CD8+ cytolytic T cells through increased activation or survival. Signals transduced by proteins such as 4-1BB, OX-40, CD27 and ICOS are regarded as especially important for survival, expansion and effector function of T cells that have initially received activating signals via the CD28 receptor [31, 32]. We previously reported increased expression of OX-40L and 4-1BBL in two colorectal tumor cell lines [44] and wanted to evaluate if the expression of other co-stimulators of CD8+ effector cells was also changed in irradiated colorectal tumor cells. For this we extended our evaluation to another TNFSF member, CD70 (CD27L/TNFSF7), and to a B7-related protein family member, ICOSL (CD275/B7-H2), and included three additional human colorectal cell lines (WiDr, HT-29 and Colo205). No increase in either CD70 (Fig. 1a) or ICOSL (Fig. 1b) was detected in HCT116 cells treated with 10 Gy of radiation. This is in contrast to the increase in 4-1BBL detected in the same cells post-IR (Fig. 1c). We also detected no increase in CD70 in any of the colorectal tumor cell lines evaluated (Fig. 1d). Furthermore, while we saw an increase in ICOSL expression in SW620 cells (0.2 %–0 Gy versus 14.10 %–10 Gy), we were unable to see a change in ICOSL expression in any of the other tumor cell lines evaluated. In addition, no increase in the expression of B7-1 (CD80) was observed following irradiation in any of the cell lines evaluated (data not shown). These data suggest that not all T cell costimulatory molecules are modulated post-IR and that the modulation of OX-40L and 4-1BBL may be selectively altered following radiation therapy of colorectal tumor cells.

### Sub-lethal irradiation of most human colorectal carcinoma cell lines enhances OX-40L and 4-1BBL expression

**Fig. 2:**
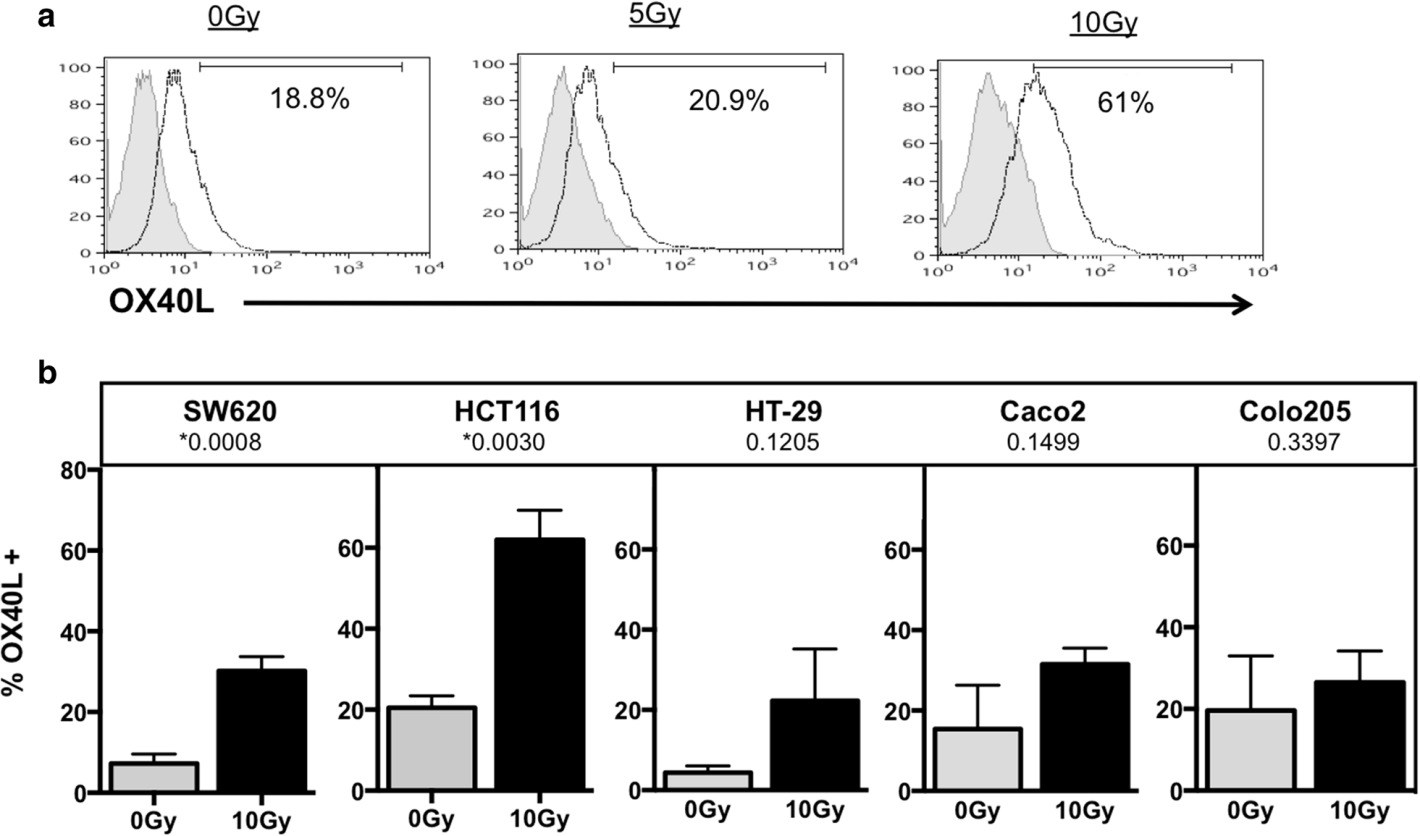
Tumor cells modulate OX-40L protein expression after treatment with radiation. **a** Irradiated (5 and 10 Gy) and non-irradiated (0 Gy) WiDr colorectal carcinoma cells were stained with PE-labeled antibody to human OX-40L. Isotype control staining is shown in gray filled histogram. OX-40L-PE positive cells are shown in solid black line histogram. **b** OX-40L expression in five additional tumor cell lines receiving 10 Gy (*black bar*) or 0 Gy (*gray bar*). *P value <0.05. *Data* *graphed* are the mean of two (Caco2), three (HCT116, Colo205, HT-29) or four (SW620) experimental repeats and *error bars* represent the SEM across the independent experiments

**Fig. 3:**
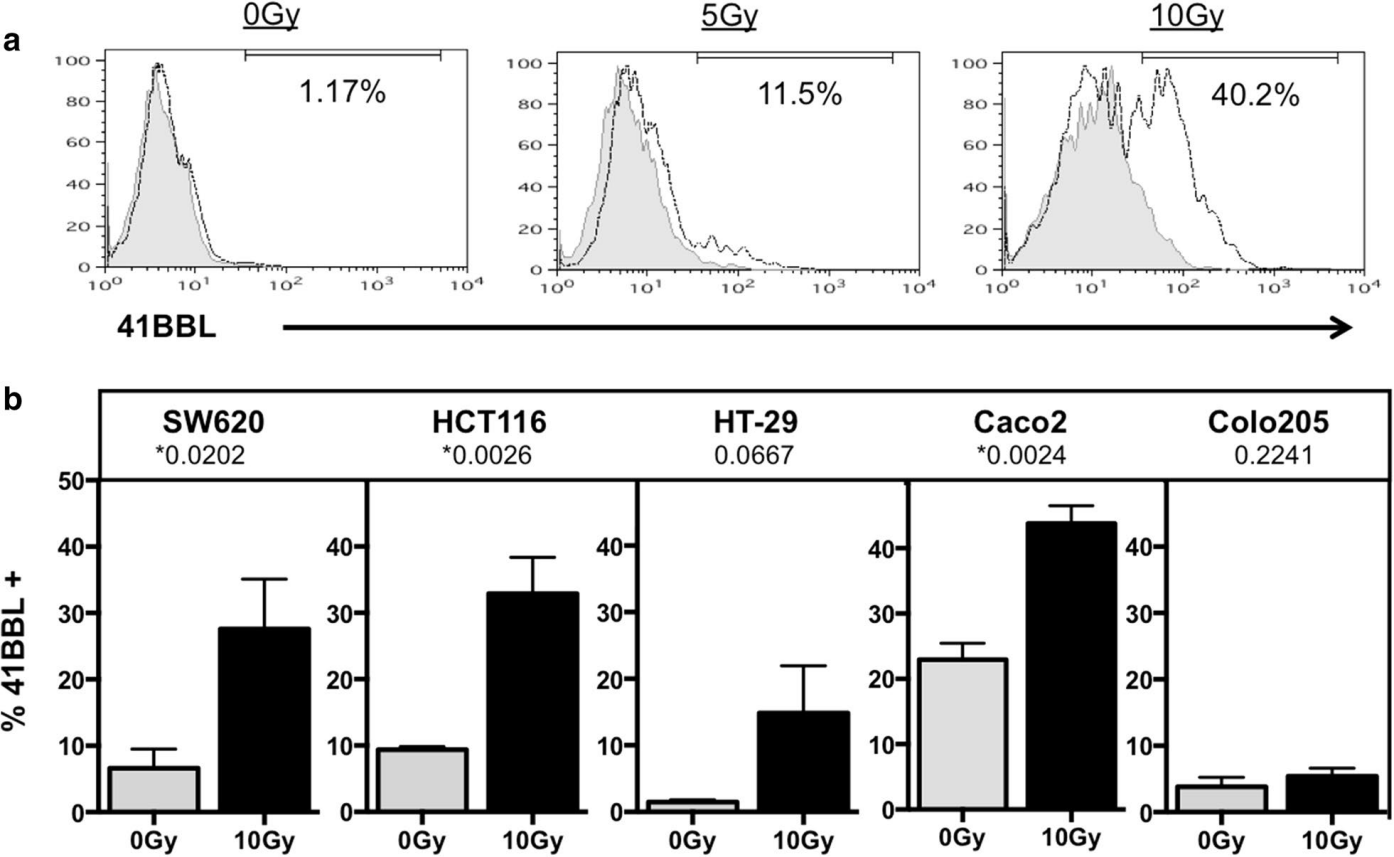
Tumor cells modulate 4-1BBL protein expression after treatment with radiation. **a** Irradiated (5 and 10 Gy) and non-irradiated (0 Gy) WiDr colorectal carcinoma cells were cultured and subsequently stained with PE-labeled antibody to 4-1BBL. Isotype control staining is shown in *gray filled histogram*. 4-1BBL-PE positive cells are shown in *solid black line histogram*. **b** 4-1BBL expression in five additional colorectal tumor cells lines receiving 10 Gy (*black bar*) or 0 Gy (*gray bar*). *P value <0.05. *Data* *graphed* are the mean of three (Colo205, HT-29, Caco2) or four (HCT116, SW620) experimental repeats and *error bars* represent the SEM across the independent experiments

To further investigate if the ability of radiation to modulate OX-40L and 4-1BBL was common among colorectal tumor cells we expanded our examination of OX-40L and 4-1BBL expression to a larger panel of human colorectal tumor cell lines (HCT116, SW620, HT-29, Caco-2, Colo205 and WiDr). Cells were irradiated, and the surface expression of these proteins was evaluated by flow cytometry after 48–72 h. We observed 18.8 % of non-irradiated WiDr cells expressed OX-40L, and this increased to 20.9 % following 5 Gy, and 61 % following 10 Gy (Fig. 2a). The average of three replicate experiments in WiDr cells revealed an average increase in expression of 44 % following radiation from 17 % in cells receiving no radiation (P = 0.0295). As reported previously, there was a significant increase in OX-40L expression in both HCT116 and SW620 (Fig. 2b) [44]. Though radiation increased the expression of OX-40L in HT-29 and Caco-2 cells repeatedly, it was not significant based on the average of replicate experiments and there was no increase in expression observed in Colo205. Staining of cells with isotype control antibody was below 5 % in all cells evaluated (data not shown). We next evaluated the surface expression of 4-1BBL in the same colorectal tumor cell lines. Figure 3a shows the level of 4-1BBL protein on the surface of untreated WiDr cells (1.17 %). The level of 4-1BBL increases following both 5 Gy (11.5 %) and 10 Gy (40.2 %) treatment. The average of three replicate experiments revealed a significant increase in expression from 4 % (0 Gy) up to 33 % following 10 Gy irradiation of WiDr cells (P = 0.0013). Expression of 4-1BBL also increased in four of the five other tumor cell lines tested (Fig. 3b) and this increase was significant in SW620, HCT116 and Caco-2 cells. Again, Colo205 cells were the exception and radiation did not increase the expression of 4-1BBL. We also evaluated the longevity of increased OX-40L and 4-1BBL and found that the elevated expression of 4-1BBL protein could still be seen 7 days post-IR in WiDr, HCT116 and SW620 cells, and the elevated expression of OX-40L was maintained in WiDr cells but not in SW620 cells (data not shown). These data suggest that expression of these proteins is modulated in most colorectal tumor cell lines by radiation, and the change can be sustained in some cases.

### Sub-lethal irradiation increases CEA and MUC-specific cytotoxic T-cell mediated killing of HCT116 and SW620 cells, but not of Colo205 cells

**Fig. 4:**
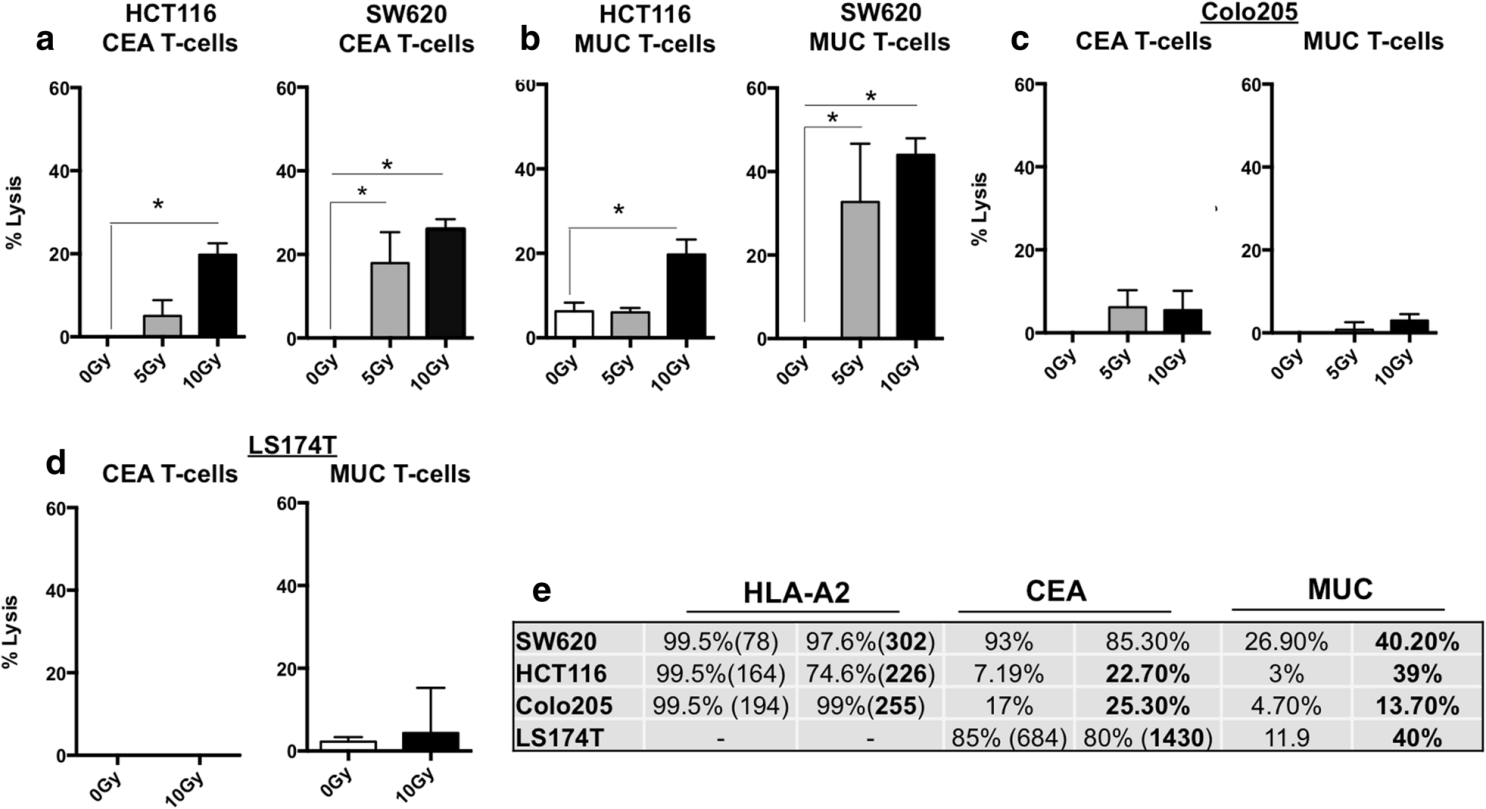
Sensitivity to CEA- and MUC-specific T-cell mediated cytolysis in irradiated colorectal tumor cells. **a** HLA-A2 positive HCT116 and SW620 cells treated in vitro with 0 Gy (*white bar*), 5 Gy (*gray bar*) or 10 Gy (*black bar*) of ionizing radiation were used as targets in a 4 h CTL cytolysis assay. At 72 h post-IR, HLA-A2 restricted CEA-specific T cells were used as effector cells at an E:T of 25:1. **b** Irradiated (5 and 10 Gy) and non-irradiated (0 Gy) HCT116 and SW620 cells were used in a 4 h lysis assays with MUC-specific T cells. At 72 h post-IR, MUC-specific T cells were used as effector cells at an E:T of 25:1 (HCT116) or 12:1 (SW620). **c** HLA-A2 positive colo205 cells were used in a cytolysis assay with either CEA-specific or MUC-specific T cells at an E:T of 30:1. **d** HLA-A2 negative LS174T cells were used in a cytolysis assay with either CEA-specific or MUC-specific T cells at an E:T of 30:1.*P value <0.05. *Error bars* indicate variability in technical replicates. Experiments were repeated at least three times with similar results. **e** Irradiated (10 Gy) and non-irradiated (0 Gy) CRC cells were cultured and subsequently stained with PE-labeled antibodies for flow cytometry to measure surface expression of HLA-A2 and TAA proteins on the surface of colorectal tumor cells. Isotype control staining of irradiated cells was less than 5 % positive. *Numbers* indicate  % of cells positive and those in *parenthesis* indicate mean fluorescence intensity (MFI) of cells expressing molecule on the cell surface 72 h post-irradiation. (*dashed line*) indicates level of detection below background. *CEA* carcinoembryonic antigen, *MUC* Mucin-1

Both HCT116 and SW620 cells are killed better post-IR by TAA-specific CTLs [26] and while both modulate surface expression of death receptors post-IR, SW620 cells have a non-functional Fas pathway while HCT116 cells are sensitive to killing through this pathway [29]. Both cells lines do, however, increase expression of OX-40L and 4-1BBL post-IR (in contrast to Colo205 cells which do not modulate either protein) (Figs. 2 and 3). We wanted to determine if increased killing could be observed against tumor cells that do not modulate the positive costimulatory molecules (Colo205), and further, to evaluate if enhanced killing occurred if a lower dose of radiation (5 Gy) was used. Human colorectal tumor cell lines HCT116, SW620 and Colo205 were irradiated with a single dose of 0, 5 or 10 Gy radiation. Following tumor cell irradiation only adherent and proliferating cells were harvested. We have previously demonstrated that tumor cells remain viable and continue to proliferate using this method [29]. At 72 h post-IR, tumor cells were evaluated in a 4 h Europium-release cell cytotoxicity assay [47] with CEA-specific CTLs. Similar to our previous observations, 10 Gy irradiated SW620 and HCT116 tumor cells were killed significantly better by CEA-specific CTLs when compared to non-irradiated tumor cells (Fig. 4a). Tumor cell lysis by CTLs could also be observed in tumor cells receiving as low as 5 Gy of radiation (17.9 % lysis of SW620 and 5 % lysis of HCT116). To evaluate if CTLs that were specific to another TAA expressed in these colorectal tumor cells would demonstrate enhanced killing of irradiated tumor targets we evaluated cytotoxic activity of mucin-1 (MUC1) specific cytotoxic T cells. MUC-specific CTLs did not lyse non-irradiated SW620 cells, however lysis increased to 32.7 and 44.1 % if tumor cells received 5 Gy (P = 0.0278) or 10 Gy (P = 0.0013) of radiation, respectively (Fig. 4b). Here, both HCT116 cells and SW620 tumor cells displayed significantly enhanced killing by MUC specific CTLs after irradiation with 10 Gy (p = 0.015 HCT116), and again killing could be observed when 5 Gy of radiation was used. Thus, these data suggest that tumor cells surviving irradiation are more susceptible to cytotoxic T-cell killing by T cells of diverse antigen specificity and at doses lower than the previously reported 10 Gy. In contrast to the killing of SW620 and HCT116 tumor cells, the percent lysis by both CEA-specific and MUC-specific T-cells was below 10 % after irradiation of Colo205 cells and untreated cells were also not killed (Fig. 4c). Thus, tumor cells that do not modulate OX-40L or 4-1BBL post-IR were also not killed by TAA-specific CTLs post-IR. As a negative control, lysis of MHC-mismatched colorectal tumor cells LS174T (CEA + MUC +/HLA-A2−) was less than 5 % at all doses of radiation in the same assay even though both CEA and MUC expression was increased (Fig. 4d). Furthermore, we observed similar enhancement of T cell survival and activation. Irradiated HCT116 and SW620 cells increased T cell survival and activation while Colo205 cells did not (data not shown).

Though HCT116 and SW620 cells modulate OX-40L and 4-1BBL post-IR and Colo205 cells do not, it is unclear what is responsible for the variable magnitude of cytolysis among the cell lines examined. These differences, however, do not appear to correlate with altered surface expression patterns of MHC-I or TAA. Specifically, HCT116 cells are killed post-IR while Colo205 cells are not (Fig. 4a, c). This occurs despite the fact that Colo205 cells express more MHC molecules per cell than HCT116 cells, as determined by both mean fluorescence intensity (MFI) (255 vs 226 respectively) and percent positive cells (74.6 vs 99.5 %) (Fig. 4e). SW620 and HCT116 cells demonstrate similarly enhanced cytolysis by CEA-specific CTLs post-IR (Fig. 4a). SW620 cells express more MHC-I (MFI 302) than HCT116, however, they actually decrease the amount of surface CEA (93 vs 85.3 %). All four cell lines were greater than 95 % positive for Pan MHC class I detected using and HLA-ABC antibody (data not shown), however LS174T were less than 5 % positive for HLA-A2. Though the impact of radiation on antigen processing and presentation remains under study, increased antigen presentation in a toleragenic or immunosuppressive environment where robust costimulation is not present could still lead to sub-optimal immune responses.

### 4-1BBL and OX-40L dual co-stimulation is required for radiation-enhanced sensitivity to CTL killing

**Fig. 5:**
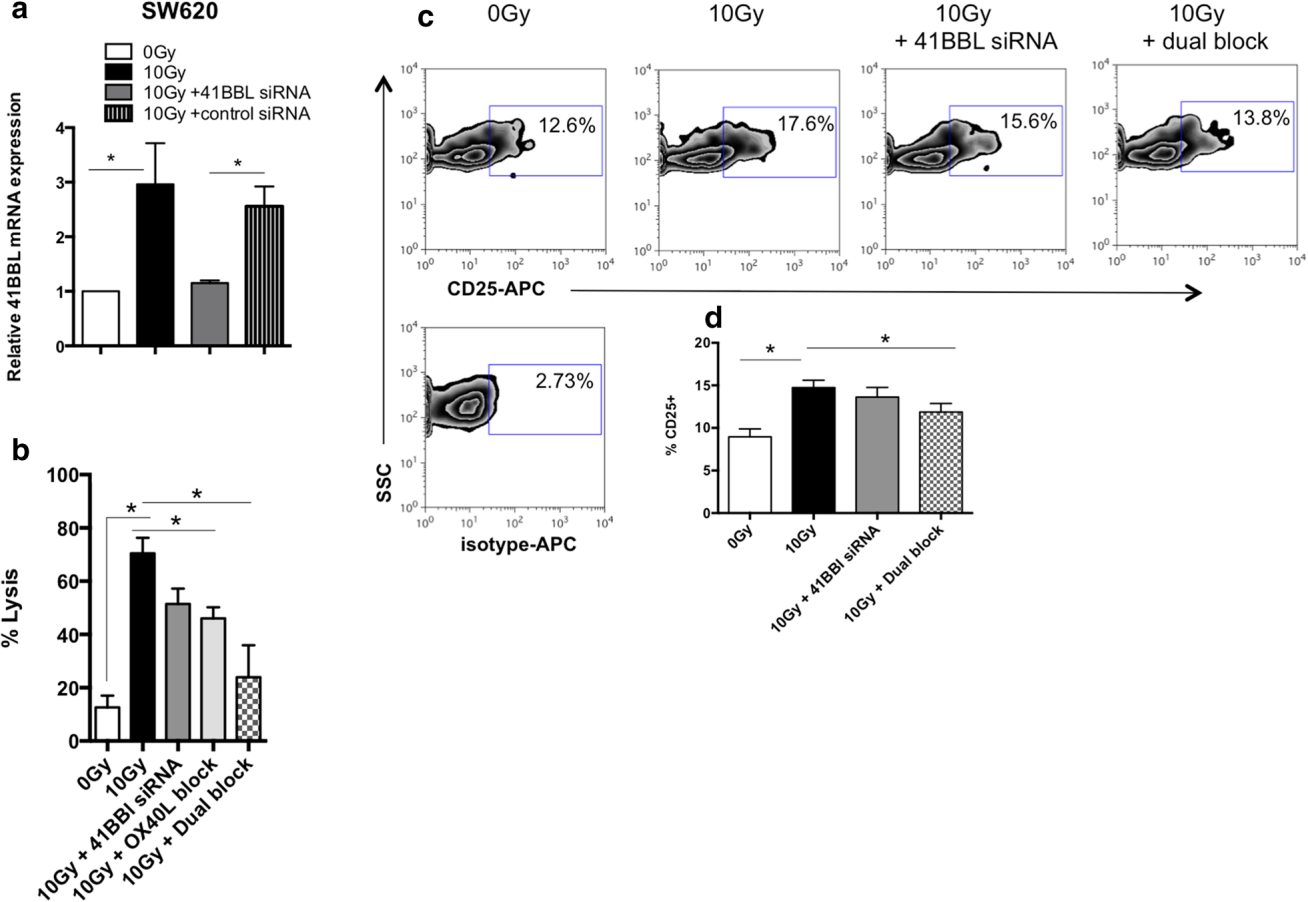
Radiation-enhanced sensitivity to T-cell mediated lysis and T-cell activation is reduced in the absence of OX-40L and 4-1BBL. **a** 4-1BBL was knocked down in tumor cells as described in “Methods” section. Briefly, 1 × 10^5^ SW620 cells were and transfected the following day with 4-1BBL siRNA or a control siRNA. 24 h post-transfection, the cells were irradiated with 10 Gy. Forty-eight hours post-IR, cells were harvested and 4-1BBL mRNA was quantified. *P value <0.05. *Data graphed* are the mean of two experimental repeats and *error bars* represent the SEM across the independent experiments. **b** 2 × 10^4^ SW620 cells were plated in 24-well plates and transfected with 4-1BBL siRNA or a control siRNA. After transfection, the cells were irradiated with 10 Gy. Forty-eight hour post-IR cells were used in a Eu-release cytotoxicity assay using CEA-specific T-cells at an E:T ration of 30:1. In the indicated groups, neutralizing antibody to human OX-40L was added to SW620 cells used in the cytolysis assay. *P value <0.05. *Error bars* indicate variability in technical replicates. Experiments repeated at least two times with similar results. **c** Forty-eight hour after cytolysis assay set-up, cells were harvested and stained for markers of T-cell activation. Flow cytometry plots showing the frequency of CD25+ cells within the CD8+ cell population after incubation with irradiated SW620 cells. **d** Data from three independent experiments is shown *graphically* and *error bars* represent the SEM across the independent experiments. *P value <0.05 between untreated versus 10 Gy treated or 10 Gy treated versus dual blockade

**Fig. 6:**
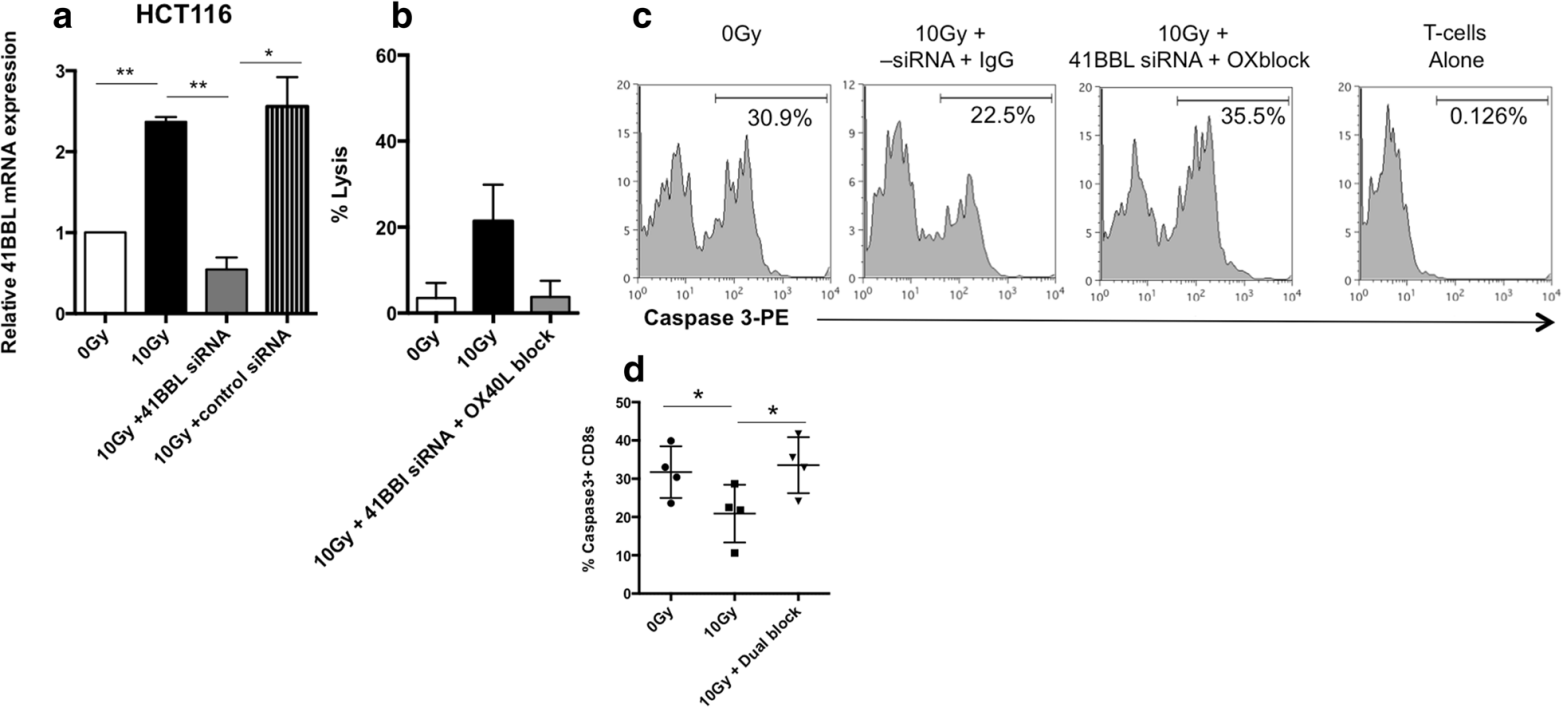
Radiation-enhanced T-cell lytic activity and radiation-enhanced T cell survival is reduced in the absence of OX-40L and 4-1BBL. **a** 4-1BBL was knocked down in tumor cells as described in “Methods” section. Briefly, 1 × 10^5^ HCT 116 cells were and transfected the following day with 4-1BBL siRNA or a control siRNA. Twenty-four hour post-transfection, the cells were irradiated with 10 Gy. Twenty-four hour post-IR, cells were harvested and 4-1BBL mRNA was quantified. *P value <0.05 and ** <0.0001. *Data graphed* are the mean of two experimental repeats and *error bars* represent the SEM across the independent experiments. **b** 2 × 10^4^ HCT 116 cells were plated in 24-well plates and transfected with 4-1BBL siRNA or a control siRNA. After transfection, the cells were irradiated with 10 Gy and used the next day in a Eu-release cytotoxicity assay as described in the “Methods” section. In the indicated group, neutralizing antibody to human OX-40L was added to tumor cells used in the cytolysis assay. *Error bars* indicate variability in technical replicates. Experiments repeated at least two times with similar results. **c** Flow cytometry histograms showing active Caspase-3 expression in T-cells incubated alone or with treated HCT116 cells for 5 h. **d** The frequency of Caspase 3+ cells within the gated CD8+ population is shown. *P value <0.05 between incubation with 10 Gy-treated tumor cells versus untreated or 10 Gy-treated cells verus 10 Gy-treated cells plus dual blockade. *Line indicates* the mean and *error bars* represent the SD across four independent experiments

In addition to delivering anti-apoptotic signals to T cells, OX-40 and 4-1BB signaling have been reported to program effector function in T cells [49–54], and result in effective anti-tumor immunity. These data indicate that T-cell effector function can be enhanced through co-stimulation of these pathways. Indeed, in the present study we detected no change in the expression of OX-40L or 4-1BBL in Colo205 cells (Figs. 2b and 3b) and, interestingly, these cells also showed no enhancement of CTL killing post-IR (Fig. 4c). These results suggest that the enhanced CTL killing of irradiated tumor cells may be due to the enhanced expression of the co-stimulatory molecules OX-40L and 4-1BBL. To further investigate whether OX-40L and 4-1BBL are involved in enhanced CTL killing of irradiated colorectal tumor cells, we performed CTL cytotoxicity assays after blocking and/or inhibiting these molecules. For these experiments, the ligand-receptor interaction of OX-40/OX-40L was blocked using neutralizing antibody against human OX-40L, and radiation-induced 4-1BBL was knocked down in tumor cells using homologous siRNA. We observed elimination of the radiation-induced increase in 4-1BBL mRNA in SW620 cells transfected with 4-1BBL-specific siRNA but not negative control siRNA (Fig. 5a). We next measured the CTL-mediated killing in tumor cells in which either 4-1BBL was knocked down using siRNA or wherein OX-40L signals were blocked using a neutralizing ab. As expected, 10 Gy of radiation enhanced CTL lysis in SW620 cells (Fig. 5b; black bars). SW620 cells displayed a reduction in CTL killing when either 4-1BBL (P = 0.0724) was knocked down by siRNA (Fig. 5b; dark gray bar) or OX-40L (P = 0.0379) signals were blocked using neutralizing antibody (Fig. 5b; light gray bar), and this was further reduced to levels similar to untreated control cells when both signals were blocked (checked bar, P = 0.0316) (Fig. 5b; checked bar). CTL cytolysis of irradiated SW620 cells was also inhibited if 4-1BB signals were blocked using a neutralizing antibody to 4-1BB (on the T-cell) in combination with the OX-40L neutralizing antibody (data not shown). Furthermore, radiation-induced activation of CD8+ CTLs, as determined by CD25 expression, was reversed when dual blockade was performed (Fig. 5c). Forty eight hours after cytolysis assay set-up 17.6 % of CD8+ cells expressed CD25 when incubated with irradiated SW620 cells as compared to 12.6 % of CD8+ cells following interaction with non-irradiated SW620 cells. When both 4-1BBL and OX-40L signals were absent only 13.8 % of CD8+ cells expressed CD25. As a positive control, CD25 expression was detected on 84 % of T-cells stimulated with phorbol myristate acetate (PMA) and ionomycin. Data from several independent experiments (Fig. 5d) depict significant changes in CD25 expression between untreated and 10 Gy treated cells (P = 0.002) as well as between 10 Gy-treated cells and 10 Gy-treated cells in the presence of dual blockade (P = 0.04). These data suggest that T cells exposed to irradiated tumor cells have improved activation. We performed similar evaluation of HCT116 cells to determine if these results could be observed in another colorectal tumor cell line. 4-1BBL mRNA was significantly reduced in cells treated with the 4-1BBL siRNA as compared to cells treated with the negative control siRNA (Fig. 6a). The enhanced cytolysis observed after irradiation with 10 Gy was reduced when both 4-1BBL was knocked down and the OX-40L blocking antibody were used in combination (P = 0.067) (Fig. 6b). Overall, these data suggest that radiation-enhanced tumoricidal activity of CTLs could be due, in part, to enhanced expression of both OX-40L and 4-1BBL and increased T cell activation via increased CD25 expression (Fig. 5c). As a component of the IL-2 receptor, CD25 it has been linked to increased survival and thus could be a contributor to the increased survival we observe following radiation treatment of tumors [44]. To determine if blocking of OX-40L and 4-1BBL signaling could reverse radiation-enhanced T-cell survival we evaluated active Caspase-3 expression in T-cells co-incubated with HCT116 tumor cells for 5 h. The flow cytometry data (Fig. 6c) are representative of three independent experiments with similar results and suggest that dual blockade on irradiated tumor cells increases the amount of T-cell death. We also evaluated the level of active caspase-3 in T-cells co-incubated with SW620 tumor cells for 18 h in blocking experiments. As before, we found active caspase-3 decrease from 31.5 % in untreated cultures down to 23.8 % in irradiated cultures. Active caspase-3 in T-cells was increased when both 4-1BBL and OX-40L signals were blocked (26 %). Data from four independent experiments (Fig. 6d) depict significant changes in the expression of Caspase 3 in T cells incubated with 10 Gy treated tumor cells as compared to both of the other groups (untreated and dual blockade). Overall, these data demonstrate that OX-40L and 4-1BBL signaling from irradiated tumor cells can enhance CTL tumoricidal activity and influence T-cell activation and survival.

## Conclusions

There is a wide array of CIT strategies under clinical investigation in combination with RT for the treatment of advanced cancers. With the exception of studies using RT for lymphodepletion prior to adoptive cell transfer [55], most clinical investigations utilize RT as an adjuvant to immune-based therapies [13–15]. These studies demonstrate enhanced immune responses, including expanded numbers of circulating anti-tumor CTLs and antibodies in treated patients. Unfortunately, these increased immune responses have not translated to a significant reduction in tumor burden often enough [14, 56, 57] and the reasons for this unexpected lack of clinical response have yet to be resolved. Detailed investigation into the molecular mechanism that results in the ability of IR to enhance anti-tumor immune responses will be required to capitalize on these biological changes and allow for additional opportunities for eliminating advanced stage cancers. More recently, modulation of tumor cells by RT has come into the spotlight and it has become clear that cells surviving radiation have an altered phenotype that can be exploited by CIT approaches [43, 58, 59]. To our knowledge, our study reports for the first time that human colorectal cells surviving radiation modulate expression of both OX-40L and 4-1BBL, and that irradiated tumor cells promote CTL tumoricidal activity related to these changes.

Our previous CTL killing assays were done using CTLs against a single antigen specificity (CEA) and a single dose (10 Gy) [26]. In our current study, killing activity of both CEA-specific and MUC-specific T cells was enhanced, suggesting that radiation-enhanced lysis is not limited to a single antigen specificity or tumor cell line (Fig. 1). We also detected enhanced susceptibility to CTL killing in tumor cells treated with a lower dose (5 Gy) of radiation. We observed enhanced CTL activity against some tumor cells treated with radiation and wondered if other aspects of T-cell biology were differentially altered in tumor cells killed (HCT116 and SW620) versus not killed post-IR (Colo205). We reasoned that T cells capable of surviving longer after interacting with tumor cells would be more likely to have productive interactions with tumor cells resulting in enhanced ability to kill tumor cells. Very few dead T cells were detected in cultures of T cells alone; however, there was an increase in T-cell death upon incubation with untreated tumor cells (Fig. 6). Death of T-cells following interaction with tumor cells has been reported by others, and is thought to be caused by tumor expressed PDL1, FasL and/or activation induced cell death (AICD) [60–62]. Surprisingly, we detected a decrease in the number of dead T cells (Fig. 6) if the tumor cells had been treated with radiation as compared to non-irradiated cells [44].

As both OX-40L and 4-1BBL have been reported to enhance T-cell survival and T-cell activation [38, 48, 63, 64], we next evaluated their expression in a panel of tumor cells. We found that five of six CRC tumor cell lines increased surface expression of both OX-40L (Fig. 2), and 4-1BBL protein (Fig. 3) after treatment with 10 Gy of ionizing radiation. Though non-treated tumor cells expressed variable amounts of both OX-40L and 4-1BBL on their cells surface, this rarely exceeded 20 % in our experiments. Salih et al. (2000) measured 4-1BBL expression on carcinoma cells and found that HCT116 cells expressed higher levels than HT-29 cells, which is in agreement with our observations in untreated tumor cells (Fig. 3b; gray bars). In contrast to OX-40L and 4-1BBL, radiation did not increase the expression of other co-stimulatory molecules evaluated in this study, including CD70, ICOSL and B7-1 (Fig. 1). The mechanism of selective gene expression is under continued investigation, and data from our lab suggests that radiation is epigenetically regulating expression of 4-1BBL and OX-40L [44]. Tumor cells irradiated in vitro may respond to radiation differently than tumors irradiated in vivo due to the influence of the microenvironment and validation of these changes in vivo is currently under study.

To determine if radiation-induced expression of OX-40L and 4-1BBL plays a direct role in enhanced tumor cell susceptibility to lysis by CTLs, we conducted CTL cytolysis experiments in combination with molecular inhibition. We found that neither knocking down 4-1BBL in tumor cells, nor inhibiting OX-40L signaling independently, completely reversed radiation-enhanced sensitivity to cytolysis (Fig. 5b). However, when both molecules were inhibited there was a more prominent loss of the radiation-enhanced killing of both SW620 and HCT116 cells by T cells (Figs. 5b and 6b), and reduced CD25 expression (Fig. 5c) and survival (Fig. 6c) of T cells. Both 4-1BBL and OX-40L signals have been reported by others to increase the production of effector molecules such as perforin and granzyme in stimulated CTLs [50, 52]. Whether irradiated tumors are impacting production or release of effector molecules from CTLs is currently under investigation. Ongoing mechanistic studies are evaluating which mechanism (increased survival or increased production/release of effector molecules or both) is primarily responsible for enhanced tumoricidal activity, as well as the relative contribution of each co-stimulatory molecule following both in vivo and in vitro tumor cell irradiation. Others have recently reported that radiation increases antigen processing and presentation pathways within tumor cells [59]. Antigen processing and presentation were not directly assessed here, however, changes in MHC-I and TAA levels in the current study did not appear to noticeably align with the changes observed in T cell activities (Fig. 4e). Given the levels of TAA and HLA-A2 among the cells lines it is difficult to imagine SW620 and HCT116, but not Colo205 cells, enhance antigen processing and presentation of CEA and MUC-1 HLA-A2 restricted peptides. Particularly when Colo205 cells and HCT116 cells express comparable amounts of CEA post-IR, and the frequency (99.5 vs 74.6 %) of HLA-A2-expressing HCT116 cells actually decreases post-IR (Fig. 4e). While the impact of radiation on antigen processing and presentation in human tumor cells remains under worthwhile investigation, it remains likely that altered antigen presentation will work in concert with other tumor changes.

The aims of this study were meant to provide data to support the growing use, and rationale application, of RT in combination with CIT. If IR-modulated expression of 4-1BBL and OX-40L is shown to play a significant role in the ability of RT to enhance effector CTL killing, this could be an alternative therapeutic approach to enhancing these important T-cell signals. This approach is particularly relevant given the severe toxicity that can occur when using agonistic antibodies. Furthermore, the use of agonist antibodies is not limited to tumor-specific T-cells and, as a result, non-tumor specific T cells can become activated and induce off-target effects. Using RT to induce these molecules, specifically on a focused target, represents a refinement in the approach by triggering these pathways in anti-tumor CTLs infiltrating irradiated tumors. Ongoing investigations include determining the impact of RT on tumor modulation in vivo more comprehensively to assess the specificity of changes on tumor cells versus other cells in the microenvironment. We have previously reported on such “immunogenic modulation” (occurring in the absence of “immunogenic cell death”) following treatment of human tumor cells with docetaxel chemotherapy [65]. Utilization of such direct tumor cell to T-cell mechanisms from phenotypically altered tumor cells that do not die post-IR, in addition to enhanced “danger” signals from dying cells, should allow for synergy resulting in a more robust anti-tumor immune attack. Ultimately, if immunomodulation of tumor cells by IR is shown to have a profound and consistent effect on CTL activity this would provide support for using IR, along with CIT strategies, specifically to enhance signals to these cells and optimize anti-tumor CTL responses.
